# Role of placental inflammatory mediators and growth factors in patients with rheumatic diseases with a focus on systemic sclerosis

**DOI:** 10.1093/rheumatology/keaa782

**Published:** 2020-12-13

**Authors:** Francesca Motta, Veronica Codullo, Véronique Ramoni, Stefania Cesari, Giuseppina Ferrario, Giacomo Fiandrino, Fausta Beneventi, Stefania Rampello, Hanna Johnsson, Carlomaurizio Montecucco, Gerard J Graham

**Affiliations:** 1 Institute of Infection, Immunity & Inflammation, College of Medical and Veterinary Life Sciences, University of Glasgow, Glasgow, UK; 2 Division of Rheumatology, Fondazione IRCCS Policlinico San Matteo and University of Pavia; 3 Unit of Anatomic Pathology, Department of Molecular Medicine, Fondazione IRCCS Policlinico San Matteo; 4 Rheumatology, University of Pavia; 5 Division of Obstetrics and Gynaecology, Fondazione IRCCS Policlinico San Matteo and University of Pavia, Pavia; 6 Division of Obstetrics and Gynaecology, ASST Papa Giovanni XXIII, Bergamo, Italy

**Keywords:** SSc, pregnancy, placenta, inflammatory cells, chemokines

## Abstract

**Objectives:**

Pregnancy in SSc is burdened with an increased risk of obstetric complications. Little is known about the underlying placental alterations. This study aimed to better understand pathological changes and the role of inflammation in SSc placentas. Leucocyte infiltration, inflammatory mediators and atypical chemokine receptor 2 (ACKR2) expression in SSc placentas were compared with those in other rheumatic diseases (ORD) and healthy controls (HC).

**Methods:**

A case–control study was conducted on eight pregnant SSc patients compared with 16 patients with ORD and 16 HC matched for gestational age. Clinical data were collected. Placentas were obtained for histopathological analysis and immunohistochemistry (CD3, CD20, CD11c, CD68, ACKR2). Samples from four SSc, eight ORD and eight HC were analysed by qPCR for ACKR2 expression and by multiplex assay for cytokines, chemokines and growth factors involved in angiogenesis and inflammation.

**Results:**

The number of placental CD3, CD68 and CD11 cells was significantly higher in patients affected by rheumatic diseases (SSc+ORD) compared with HC. Hepatocyte growth factor was significantly increased in the group of rheumatic diseases patients (SSc+ORD) compared with HC, while chemokine (C-C motif) ligand 5 (CCL5) was significantly higher in SSc patients compared with ORD and HC. CCL5 levels directly correlated with the number of all local inflammatory cells and higher levels were associated with histological villitis.

**Conclusions:**

Inflammatory alterations characterize placentas from rheumatic disease patients and could predispose to obstetric complications in these subjects.

**Rheumatology key messages**

- Placental leukocytes are more numerous in rheumatic diseases, with a possible role in obstetric complications.
- HGF placental levels are higher in rheumatic diseases than in controls and may promote placentation.
- CCL5 expression is higher in SSc placentas and this supports its pathogenetic role.

## Introduction

Patients with rheumatic diseases (RD), especially connective tissue diseases, are at increased risk of obstetric complications and have historically been advised against pregnancy. In recent years, contraindications have been revised in light of new knowledge of the pathogenesis of the complications and of therapies for their management [1–4].

Most studies examining fetal outcome and placental changes in RD concern SLE and APS. Preterm birth, intrauterine growth restriction (IUGR) and preeclampsia are frequent complications in SLE [5] and are associated with trophoblast alterations, villitis, vasculopathy and a high number of inflammatory cells [6, 7]. In APS the higher risk of abortion, stillbirth, IUGR and preterm birth [8] is associated with trophoblast alterations, infarction and a higher number of placental inflammatory cells [9, 10]. In chronic arthritis, a slightly increased risk of spontaneous abortion or preterm birth compared with healthy population has been described and a lot of studies have been performed with respect to therapy [11], but no histological analysis of the placenta has been conducted so far.

An Italian multicentre study showed that women with SSc have a higher than normal risk of IUGR, preterm delivery and very low birth weight babies [12]. In a case series of 13 SSc patients [13], five showed decidual vasculopathy, associated with fetal death in four cases. The vessels had increased number of perivascular macrophages, immunoglobulin deposits and CD4 lymphocytes compared with healthy controls. A study of three cases [14] described decidual vasculopathy, villous hypovascularity, stromal fibrosis, increased syncytiotrophoblast knotting and infarcts in the placentas of SSc patients compared with healthy controls. Immunohistochemical analysis revealed increased staining for VEGF, VEGF receptor 2, connective tissue growth factor and α-smooth muscle actin in myofibroblasts in SSc patients, as signs of altered vascular remodelling and fibrosis.

We are particularly interested in the role of the atypical chemokine receptor 2 (ACKR2), which does not signal in response to chemokines, but internalizes ligand and targets it for intracellular degradation, acting as a chemokine ‘scavenger’ [15]. It is highly expressed in trophoblasts and may be important in reducing the risk of inflammation-related miscarriage, minimizing inflammatory chemokine exchange between mother and fetus [16]. ACKR2 knock out mice have fetal loss if infused with antiphospholipid antibodies or lipopolysaccharides [17]. Furthermore, ACKR2 levels are higher in the peripheral blood mononuclear cells (PBMCs) of patients with SSc compared with healthy controls [18].

The aim of our study was to analyse the histopathological placental features of a cohort of SSc patients, with a focus on the role of inflammation in the pathogenesis of obstetric complications and to determine whether placental ACKR2 might have a role in it.

## Methods

### Patients

Patients attending the Rheumatology Unit of the IRCCS Policlinico San Matteo’s Foundation in Pavia, Italy, who fulfilled the 2013 European League Against Rheumatism/American College of Rheumatology classification criteria for SSc [19] and who consecutively became pregnant between 2013 and 2018, were enrolled in this prospective study. Pregnant patients with other RD (ORD) classified according to the current classification criteria [20–23] were enrolled as the first control group and healthy pregnant women followed at the Gynaecology and Obstetrics Unit formed the second control group (healthy controls, HC). Patients for comparison groups were consecutively enrolled if matched to SSc patients by age, body mass index and week of delivery, with a ratio of 1:2:2. Patients were followed up by the same physicians during pregnancy. Organ involvement was evaluated according to the presence of signs and symptoms of disease at the visits and imaging data. Pulmonary involvement was recorded if the chest X-ray, high resolution CT scan of the thorax, pulmonary function tests or echocardiography had previously given an indication of interstitial or vasculopathic lung disease. Laboratory tests, including autoantibodies, were evaluated using commercially available kits.

This study was carried out in accordance with the Declaration of Helsinki. The local ethics committee has approved the research protocol and all patients provided their written informed consent to use their placentas in the study.

### Macroscopic and histopathological analysis

Placentas were weighed and underwent macroscopic examination. Full thickness samples were obtained, fixed in 10% buffered formalin and embedded in paraffin. Sections (3 µm) were stained with haematoxylin, eosin and Masson’s trichrome for histopathological examination according to the most recent guidelines [24] by an expert pathologist who was blind to sample classification.

### Immunohistochemistry

Paraffin-embedded full thickness placental samples were sliced into 3 µm sections, dewaxed and heated in 0.01 M pH 6 sodium citrate buffer for antigen retrieval. After blocking endogenous peroxidase activity and non-specific binding, the sections were incubated overnight with the following primary antihuman antibodies: mouse monoclonal anti-CD3 (F7.2.38, 1:70, Dako, Glostrup, Denmark), mouse monoclonal anti-CD20 (L26, 1:126, Dako), mouse monoclonal anti-CD68 (PG-M1, 1:30, Dako), rabbit monoclonal anti-CD11c (EP1347Y, 1:500, Abcam, Cambridge, UK) and rabbit polyclonal anti-ACKR2 (1:400, Sigma-Aldrich, St Louis, MO, USA). Sections were then incubated with the appropriate chromogenic secondary antibody (ImmPRESS Polymer Detection Kit, anti-rabbit and anti-mouse, Vector Laboratories, Burlingame, CA, USA). The immunoreactivity was developed using 3,3′-diaminobenzidine tetrahydrochloride (Vector Laboratories) as chromogen. Isotype-matched control antibodies were included as a negative control and tonsil sections as a positive control. The sections were observed under a light microscope (Olympus BX43, Olympus, Tokyo, Japan) and photographed by digital camera (DP22 using Olympus Cell Sense Entry 2.2 for imaging acquisition).

### Analysis of immunostaining

The immunostaining for CD3, CD20, CD11c and CD68 was assessed as follow. Photographs were taken of 10 random fields (×40 magnification) along the sections and representative of all placental layers. Stained cells were counted by two blinded observers and normalized to the tissue area. The percentage of stained area was assessed in sections stained for ACKR2 and with Masson’s thrichrome. ImageJ 2.0 software was used to measure stained area and total area of tissue represented in the fields examined.

### Real-time quantitative polymerase chain reaction

Random parenchymal biopsies were performed in half of the samples and stored in RNAlater (Thermo Fisher Scientific, Waltham, MA, USA) at −80°C. To extract RNA, samples were lysed and homogenized in β-mercaptoethanol and RLT buffer by shaking with steel beads in a Tissue Lyser LT (Qiagen, Valencia, CA, USA). RNA was then extracted and purified from the fluid phase using the RNeasy Mini extraction kit (Qiagen). Purified RNA was converted to cDNA using the high capacity RNA to cDNA kit (Thermo Fisher Scientific). Samples were tested in triplicate and qPCR for ACKR2 was performed as previously described [25]. ACKR2 transcript levels were normalized to TATA-binding protein. The samples were run on a QuantStudio 7 flex machine (Thermo Fisher Scientific).

### Protein extraction and multiplex cytokine assay

Placental samples were suspended in tissue extraction buffer (homemade with 100 mM pH 7.4 Tris, 150 mM NaCl, 1 mM EGTA, 1 mM EDTA, 1% Triton X-100, 0.5% sodium deoxycholate and protease inhibitors), homogenized and the concentration of total proteins in the supernatant was determined by Pierce BCA Protein Assay Kit (Thermo Fisher Scientific). Protein concentration in the samples was normalized to the sample with the lowest concentration. Samples were analysed as per protocol using a 30-Plex bioassay (Thermo Fisher Scientific) measuring interleukin (IL)-1β, IL-1ra, IL-2, IL-2R, IL-4, IL-5, IL-6, IL-7, IL-8, IL-10, IL-12 (p40), IL-13, IL-15, IL-17, TNF-α, IFN-α, IFN-γ, GM-CSF, G-CSF, chemokine (C-C motif) ligand (CCL)2, CCL3, CCL4, CCL5, chemokine (C-X-C motif) ligand (CXCL)9, CXCL10, CCL11, VEGF, fibroblast growth factor, hepatocyte growth factor (HGF) and epidermal growth factor.

### Statistical analysis

For all statistical tests, non-parametric data were analysed using the Mann–Whitney *U*-test and parametric data using Student’s unpaired *t*-test. For multiple comparisons, a Kruskal–Wallis correction was applied to the test. To detect significant correlation between variables, Spearman’s correlation coefficient was used, where *r* = 1 denotes a perfect positive correlation and *r* = −1 a perfect negative correlation. *P* < 0.05 denotes significant differences. Comparisons were also made between each of the following groups: SSc, SLE, UCTD, defined connective tissue disease (SSc+SLE+Sjögren’s syndrome) patients; SSc patients with obstetric complications, SSc patients without obstetric complications, SSC+ORD patients with obstetric complications, SSc+ORD patients without obstetric complications, HC, all complicated pregnancies, patients who had preeclampsia, patients with poor fetal outcome (IUGR, small for gestational age, death), patients with preterm birth, patients with premature rupture of membranes, all non-complicated pregnancies.

Statistical analyses were performed using Prism 8.0.2 for Macintosh (GraphPad Software Inc., La Jolla, CA, USA).

## Results

### Main clinical features of the study patients

A total of eight patients affected by SSc, 16 with ORD and 16 HC were enrolled in the study. All SSc patients but one took low dose acetylsalicylic acid during pregnancy and one patient took prednisolone 4 mg daily in addition. Their main clinical and pregnancy-related features are shown in Table 1.

**Table 1:** Characteristics of SSc patients

Patient	Disease subset	Duration of disease, years	mRSS	Autoantibodies	Internal organ involvement	Therapy before/during (b/d) pregnancy	**BMI, kg/m^2^**	Comorbidities and risk factors	**Age at conception, years**	Gestational week at delivery	Obstetric complications	Newborn weight, g	Placental characteristics (descriptive)
1^a^	lc-SSc	3	2	ACA	None	b and d: levothyroxine, ASA	21.5	Gestational hypothyroidism	34	38 + 6	PROM	3170	Dystrophic calcifications, mild acute chorioamnionitis
2^a^	lc-SSc	1	4	ACA	Gastrointestinal	b: prostanoids. d: MPD 4 mg	23.8	—	38	36	HELLP, preterm birth	2660	Hypoxic hypervascularization, syncytial knots, decidual inflammation
3^a^	dc-SSc	12	13	Anti-Scl70	None	b: bosentan, prostanoids. d: CCB, ASA	28.1	—	31	40	—	3175	Focal subchorial fibrin deposits
4^a^	dc-SSc	1	3	Anti-Scl70	None	b and d: ASA	22.6	—	33	40	Preeclampsia	3690	Mild acute chorioamnionitis, low grade chronic villitis, few dystrophic calcifications
5	dc-SSc	1	2	Anti-Scl70	None	b and d: ASA	23.1	—	29	36 + 4	Preterm birth	2800	Dystrophic calcifications, mild acute chorioamnionitis, fibrin deposits
6	dc-SSc	4	6	Anti-Scl70	None	b: prostanoids. d: levothyroxine, ASA	26	Hashimoto’s thyroiditis	29	40	—	3140	Rare avascular villi, perivillous and villous fibrin deposits, focal chronic villitis
7	dc-SSc	2	2	Anti-Scl70	None	b: CCB d: LMWH, ASA	25.5	—	35	26	Preterm birth, IUGR, HELLP	511, SGA	Villous haemorrhage, areas of infarction, decidual arteriopathy, mural thrombi
8	dc-SSc	7	12	Anti-Scl70	Gastrointestinal, cardiopulmonary	b: CCB d: ASA	27	—	34	30 + 6	Preterm birth, neonatal death	1428	Chronic decidual inflammation, decidual arteriopathy

a Patient with both paraffin and frozen samples. ASA: acetylsalicylic acid 100 mg; CCB: calcium channel blockers; dc-SSc: diffuse cutaneous SSc; HELLP: haemolysis, elevated liver enzymes, low platelets; IUGR: intrauterine growth restriction; lc-SSc: limited cutaneous SSc; LMWH: low molecular weight heparin; MPD: metylprednisolone; mRSS: modified Rodnan skin score; PROM: premature rupture of membranes; SGA: small for gestational age.

The patients affected by ORD included 10 patients with UCTD, characterized by presence of autoantibodies and arthritis or cytopenias, none satisfying Very Early Diagnosis of SSc (VEDOSS) classification criteria, three with SLE, two with idiopathic juvenile arthritis and one patient with Sjögren’s syndrome (SjS). Their main clinical and pregnancy-related features are shown in Supplementary Tables 1 and 2, available at *Rheumatology* online.

### Macroscopic and histopathological findings

No macroscopic placental differences (dimension, weight) were observed between groups. At histological examination, no significant difference (*P* > 0.05) was found between groups, or between the HC *vs* the SSc patients and *vs* the RD patients (SSc+ORD) regarding the presence of deciduitis, villitis, materno-fetal inflammation, placental abruption, vascular alterations or fibrin deposits. The latter was examined both by histopathological examination and by Masson’s trichrome staining. The histopathological data did not show any correlation or association with disease-related features as disease subset, disease duration, modified Rodnan skin score (mRSS) and organ involvement.

### Inflammatory cells within placentas

**Fig. 1:**
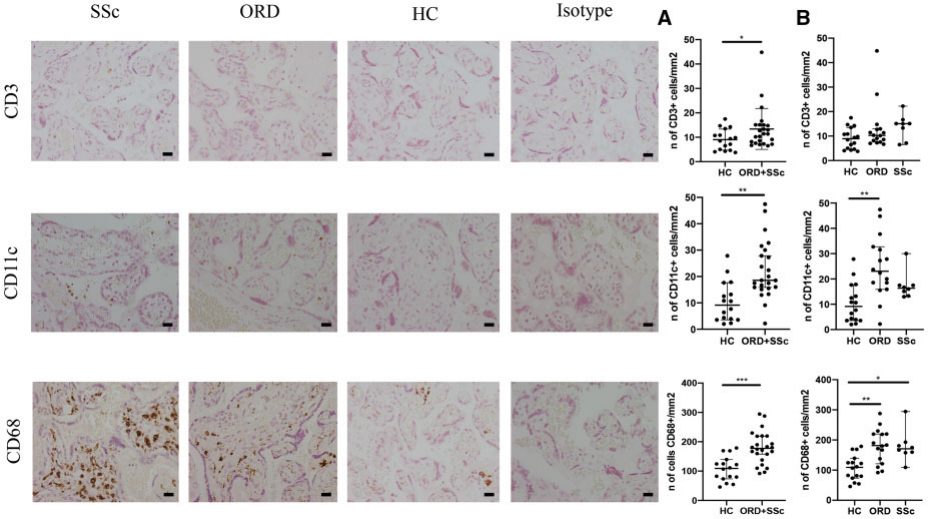
Immunohistochemical analysis (**A**) CD3, CD11c and CD68 expression in placentas from patients with SSc, other rheumatic diseases (ORD) and healthy controls (HC). Scale bar: 20 μm. (**B**) Median and 95% CI of CD3+, CD11c+ and CD68+ cell expression per mm^2^ in HC compared with rheumatic disease patients (ORD+SSc). (**C**) Median and 95% CI of CD3+, CD11c+ and CD68+ cell expression per mm^2^ of HC, ORD and SSc groups. *P <0.05, **P <0.001, ***P <0.0001

**Fig. 2:**
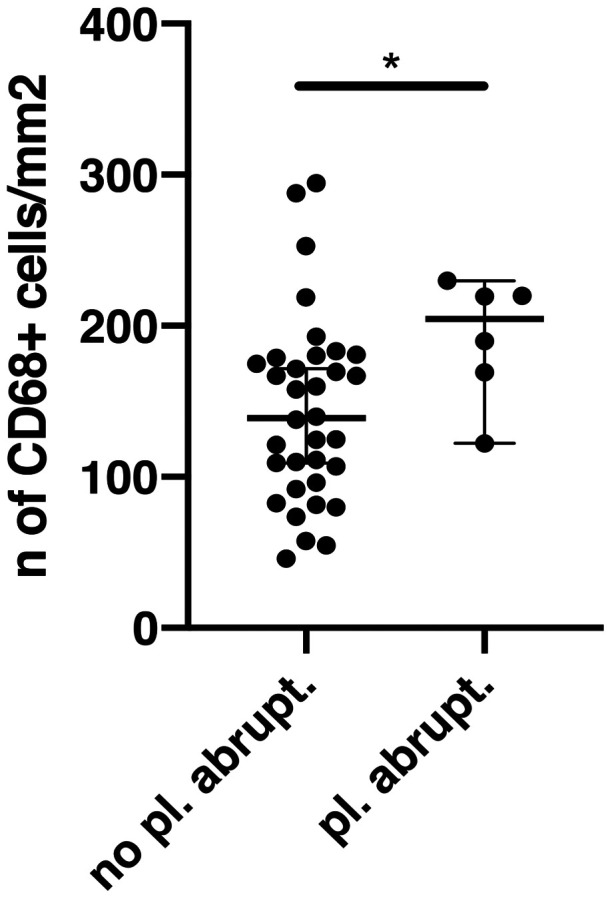
Placental macrophages in relation to placental abruption The number of CD68+ cells/mm^2^ in placentas from patients with and without placental abruption (pl. abrupt.). *P <0.05.

The number of placental CD3 and CD11c+ cells found by immunohistochemistry was significantly higher in patients affected by RD (SSc+ORD) compared with HC. The SSc group alone did not statistically differ from the ORD group nor the HC, possibly due to a smaller sample size. The number of placental CD68+ cells was significantly higher in both the SSc and ORD groups compared with HC (Fig. 1). Patients with histological evidence of placental abruption had a higher number of placental CD68+ cells, regardless of diagnosis (Fig. 2).

The number of CD20+ cells was not statistically different between the groups (Supplementary Fig. S1, available at *Rheumatology* online), even though there was a trend towards higher numbers in the SSc+ORD group (*P* = 0.058).

Similar results were obtained comparing SSc patients and HC: CD3+ (*P* < 0.05), CD11c+ (*P* < 0.05) and CD68+ (*P* < 0.01) cells were significantly higher in the first group, while CD20+ cell number was not different.

### Placental ACKR2 expression and transcription

There was strong staining of ACKR2 in all sections and there was no difference in the stained area between the groups (SSc *vs* ORD *vs* HC, SSc *vs* HC, SSc+ORD *vs* HC). Real-time quantitative polymerase chain reaction (RT-qPCR) analysis showed very high transcript levels in all groups, without significant differences between them. Moreover, ACKR2 expression and transcription levels did not correlate with any clinical (disease subset, disease duration, mRSS, internal organ involvement) or obstetric variable (presence of complications, week of delivery, presence of histological alterations as above detailed).

ACKR2 transcript levels correlated with the percentage of stained area in immunohistochemistry (Supplementary Fig. S2, available at *Rheumatology* online), indicating concordance with protein expression.

### Inflammatory mediators and growth factors in placenta

**Fig. 3:**
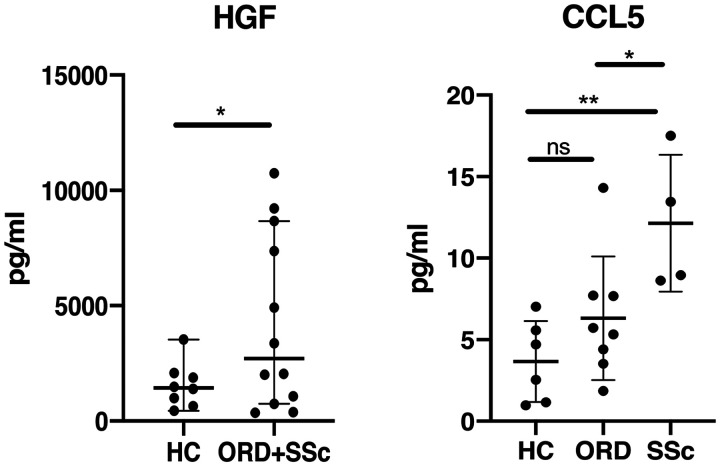
HGF and CCL5 placental levels Levels of the hepatocyte growth factor (HGF) and of chemokine (C-C motif) ligand 5 (CCL5) in healthy controls (HC), patients with other rheumatic diseases (ORD) and SSc. *P <0.05, **P <0.01, ns: not significant.

We measured levels of a broad range of inflammatory mediators and growth factors in the placentas from four SSc patients (two dc-SSc, two lc-SSc), eight patients affected by ORD and eight HC. Only those molecules showing significant differences between groups were considered in further analyses and in clinical correlates. Specifically, HGF was significantly increased in RD patients (SSc+ORD) compared with HC (*P* < 0.05) and CCL5 was significantly higher in SSc patients compared with ORD (*P* < 0.05) and with HC (*P* < 0.01) (Fig. 3).

When analysing SSc *vs* HC group, HGF levels were not different (*P* > 0.05), while CCL5 levels were significantly higher (*P* < 0.01).

**Fig. 4:**
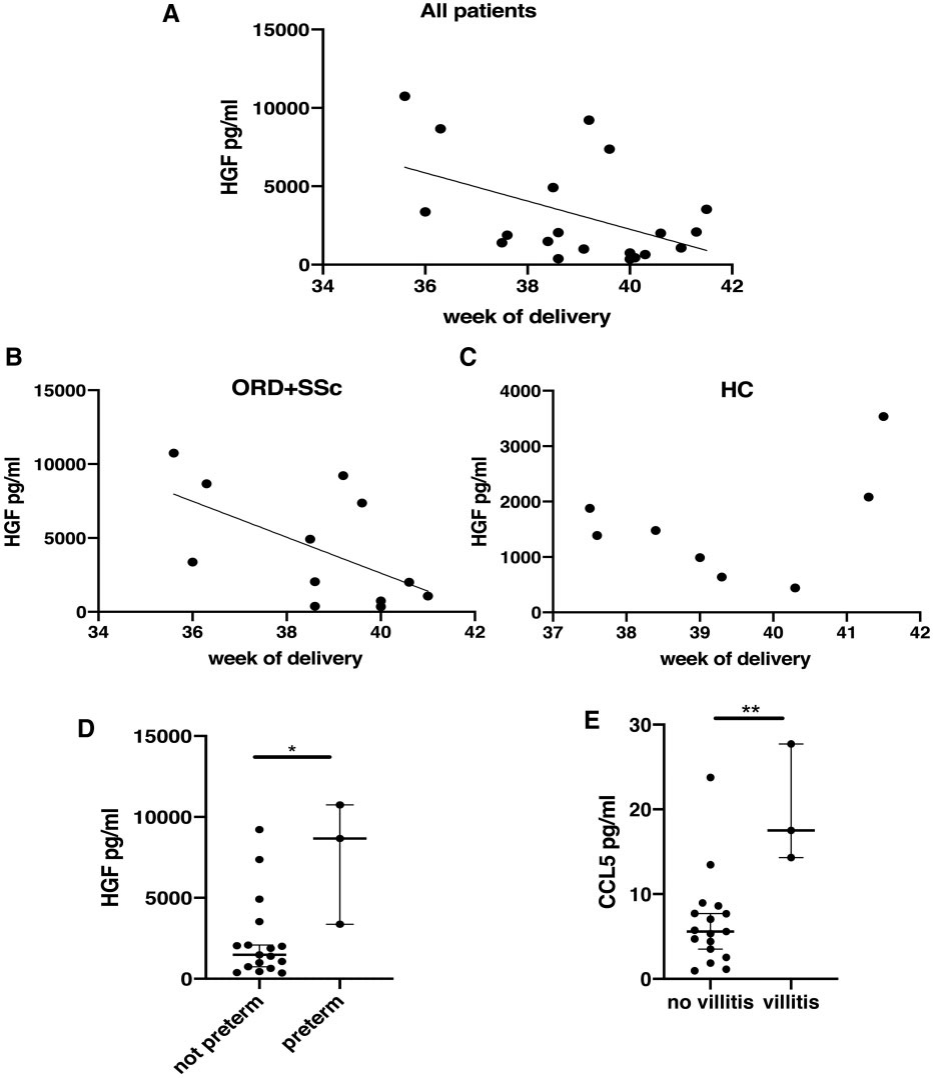
HGF/CCL5 levels and obstetric complications (**A**) Considering all patients, levels of hepatocyte growth factor (HGF) inversely correlated with the gestational week at delivery (*r* = 0.47, *P* < 0.05). (**B**, **C**) The inverse correlation is maintained in the rheumatic diseases group [other rheumatic diseases (ORD)+SSc; *r* = 0.5, *P* < 0.05] (**B**), but not in the healthy controls (HC) group (*P* > 0.05) (**C**). (**D**) Differences in HGF levels in patients with and without preterm delivery. (**E**) Chemokine (C-C motif) ligand 5 (CCL5) levels in patients with and without histological villitis. *P < 0.05, **P < 0.01.

HGF levels inversely correlated with the gestational week at delivery (Fig. 4A) and when the disease groups were analysed separately, a significant inverse correlation was seen in the rheumatic disease patients group (SSc+ORD), but not in HC (Fig. 4B and C). The same was detected for placental weight, which inversely correlated with HGF in patients affected by RD (Supplementary Fig. S3, available at *Rheumatology* online). Accordingly, HGF levels were higher in patients with preterm delivery, regardless of the diagnosis (Fig. 4D). Higher levels of placental CCL5 were associated with histological villitis (Fig. 4E).

No clear associations were seen between HGF, CCL5 and disease-related clinical features (disease subset, disease duration, mRSS, internal organ involvement), while direct significant correlation was noted between CCL5 and the number of all inflammatory cells considered in immunohistochemistry. Moreover, the number of CD3+ cells directly correlated with the number of CD20+ and CD11c+ cells. The number of CD68+ cells directly correlated with the number of CD11c+ cells and with decidual HGF levels (see Table 2 for descriptive statistics presented as *r*-values).

**Table 2:** Correlations between the number of inflammatory cells, ACKR2 transcript, HGF and CCL5 levels

	CD3						
CD3	1	CD20					
CD20	0.41^**^	1	CD11c				
CD11c	0.37^**^	0.10	1	CD68			
CD68	0.25	0.19	0.49^***^	1	ACKR2		
ACKR2	−0.01	0.1	−0.4	−0.5	1	HGF	
HGF	0.1	−0.3	0.2	0.5^**^	−0.2	1	CCL5
CCL5	0.3^*^	0.4^**^	0.4^*^	0.3^*^	−0.2	0.02	1

Correlation coefficients obtained from Spearman tests. ^*^P <0.05, ^**^P <0.01, ^***^P <0.001. ACKR2: atypical chemokine receptor 2; CCL5: chemokine (C-C motif) ligand 5; HGF: hepatocyte growth factor.

### Comparisons between distinct rheumatic diseases

As the ORD group included heterogeneous RD, we analysed if any differences in placental leukocytes, inflammatory mediators or growth factors, or ACKR2 levels could be detected among them. In particular, we considered SSc *vs* SLE patients, SSc *vs* UCTD patients, SLE *vs* UCTD patients and UCTD *vs* defined connective tissue disease (SSc+SLE+SjS). CD20+ cells were higher in placentas from defined connective tissue diseases compared with UCTD (*P* < 0.01). No other significant findings were observed between groups, except from a trend toward higher placental CCL5 levels (*P* = 0.06) in SSc compared with UCTD patients.

### Comparisons between successful and complicated pregnancies

We investigated if any distinctive alteration could be found in patients with obstetric complications. Therefore, we considered sub-groups of patients and analysed if differences could be detected in placental CD3+, CD20+, CD11c+, CD68+ cells, ACKR2 expression and transcription, inflammatory mediators and growth factors. No significant findings have been detected, except from a trend toward higher placental CD68+ cells (*P* = 0.07) and HGF (*P* = 0.06) in preterm placentas and to higher CCL5 in patients with preeclampsia (*P* = 0.07).

## Discussion

In this study we analysed how inflammation might play a role in obstetric complications that frequently occur in the pregnancies of patients affected by RD, in particular SSc. To our knowledge, this is the largest cohort thus far analysed in SSc.

We found that patients with RD had higher numbers of placental leukocytes, specifically T lymphocytes (CD3+ cells), antigen-presenting cells (APCs, CD11+ cells) and macrophages (CD68+ cells), compared with HC.

Our results are in line with and reinforce previous literature showing an increased number of placental leukocytes in these patients [6, 7, 9, 10]. This has been associated with obstetric complications such as IUGR, preeclampsia, fetal death and preterm delivery [26–28]. Placental macrophage infiltration might play a role in reducing trophoblastic invasion, in placental abruption [9, 29] and in preterm labour [30]. In addition, an association between high maternal serum and placental concentrations of M-CSF with IUGR [31] and preeclampsia [32] has been reported. Other evidence suggests that placental T cell infiltration and imbalance are important in the aetiopathogenesis of preeclampsia [33]. In our population a higher number of placental macrophages was associated with placental abruption and a trend towards higher CD68+ cells in preterm placentas was shown, regardless of diagnosis of rheumatic diseases. No other significant association was found between inflammatory cell numbers and obstetric complications, considering SSc patients, RD patients or all complicated pregnancies regardless of diagnosis. It is possible that with our small population we did not have enough statistical power to detect more subtle differences in other leucocyte populations among patients with and without obstetric complications (and in sub-groups of rheumatic diseases patients). Therefore, we can only speculate that SSc patients, and in general RD women, may be more predisposed to obstetric complications due to the development of placental inflammatory alterations.

The proangiogenic factor HGF [34] was higher in patients with RD (SSc+ORD) compared with HC. In placenta, HGF is produced by stromal cells of the villous mesenchyme and stimulates trophoblast invasion in the decidua [35]. Its levels are reduced in hypoxic conditions and in patients with preeclampsia [36]. It might be speculated that patients with RD need higher levels of HGF to promote trophoblast invasion and placentation. In support of this, in our population HGF levels inversely correlated with gestational week and placental weight in patients with RD but not in controls, suggesting an important role of this factor in women affected by autoimmune diseases, with higher levels in early stages when placenta is still developing and lower values in the end stages of pregnancy.

An important insight provided by our study concerns CCL5, which was significantly higher in placenta from patients with SSc compared with ORD and HC, with no difference between the latter two groups and which appeared to be related to villitis and to preeclampsia, regardless of rheumatic disease, although the latter association did not reach full statistical significance. These may indicate a disease specific role of this chemokine in SSc. CCL5 mediates trafficking and activation of several immune cells [37]. An association has been demonstrated between a specific polymorphism of the gene coding for CCL5 and susceptibility for SSc [38]. CCL5 has been implicated in the pathogenesis of perivascular inflammation, vascular dysfunction [39, 40], hepatic and renal fibrosis [41–43], and myocardial remodelling [44]. Furthermore, CCL5 is highly expressed in the skin of patients with SSc, while no expression has been found in the skin of controls [45]. Specifically, CCL5 is highly expressed in skin in early SSc, as are CCL2, CCL3, CCL4 and CX3CL1. In advanced stages CCL7 and CXCL10 predominate [46]. The early expression of CCL2, CCL3 and CCL5 is also observed in a mouse model of scleroderma, with a subsequent rapid reduction of CCL5 and maintained high expression of CCL2 and CCL3 [47]. Another study showed that CCL2, CCL3 and CCL5 were significantly higher in serum of patients with SSc than in controls and therapy with prostaglandins down-regulated CCL2 and CCL5, suggesting an effect of vasodilator therapy on inflammation in SSc [48]. Considering the placenta as a new organ, with possible gradual involvement by the disease, CCL5 could be a key regulator of the pathological process. Through its chemoattractive activity it could promote the formation of a placental inflammatory infiltrate, and in fact in this study we have shown a correlation between CCL5 levels and leukocytes infiltration. Moreover, it could be a key factor in the development of vascular alterations and, in the subsequent stages, of fibrosis.

We did not find different levels of transcription or expression of placental ACKR2 in SSc or ORD compared with HC. In a previous study, ACKR2 levels were higher in PBMCs of SSc patients compared with controls [18]. Furthermore, ACKR2 was elevated in PBMCs and synovial tissue of patients with inflammatory arthropathies [49]. In our population, PBMCs from pregnant patients were not always available and thus we could not perform a group analysis, but it would be interesting in future studies to compare PBMCs and placental levels of ACKR2. A possible explanation for similar ACKR2 levels in our groups could be that ACKR2 is strongly expressed in placenta and in our patients its immunomodulatory role was sufficient to control the inflammation induced by the inflammatory cells present in the tissue. In fact, the number of leukocytes, although higher in patients with RD, was not associated with obstetric complications, except from placental abruption. The only ACKR2 ligand found to be elevated was CCL5 in patients with SSc, underlining a prominent activity of this chemokine in these patients.

In conclusion, there is increased placental leucocyte infiltration in patients with RD and this may contribute to the risk of complications. High HGF levels could represent a protective mechanism for an adequate placentation. In SSc, CCL5 might be a key factor, with a role in chemotaxis, vascular remodelling and fibrosis development.

This could be considered a pilot study and a larger population of SSc and RD patients should be enrolled in order to improve statistical power, perhaps in a multicentric study. The detection of defined inflammatory alterations could help in understanding the pathogenesis of the poor outcomes affecting SSc and RD pregnancies. Moreover, an analysis of inflammatory features in relation to therapy could be performed, to detect if low dose corticosteroids, hydroxychloroquine, other immunosuppressants or anti-platelet agents could have a role in SSc and RD placental alterations and in specific obstetric complications. In addition, a comparison between alterations in placental tissue and peripheral blood could lead to the detection of serum markers predictive of higher-risk pregnancies, easy to detect at early stages, when a timely personalized pharmacological intervention may be performed to prevent complications.

## Supplementary Material

keaa782_Supplementary_Data
