# Supplementary material for: Role of placental inflammatory mediators and growth factors in patients with rheumatic diseases with a focus on systemic sclerosis
Source: Rheumatology (Oxford). 2020 Dec 13;60(7):3307–16. doi: 10.1093/rheumatology/keaa782 (PMC8516508; doi:10.1093/rheumatology/keaa782)
Supplement: keaa782_Supplementary_Data [file keaa782_Supplementary_Data.zip › rhe-20-1525-File009.doc]

**Supplementary table 1. Characteristics of patients affected by other rheumatic diseases (ORD).**

| Patient | Disease | Duration of disease (years) | Autoantibodies | Organ involvement | Therapy before/during (b/d) pregnancy | BMI | Comorbidities and risk factors | Age at conception | Gestational week at delivery | Obstetric complications | Newborn weight (gr) | Placental characteristics (descriptive) |
| --- | --- | --- | --- | --- | --- | --- | --- | --- | --- | --- | --- | --- |
| 1* | UCTD | 3 | ANA 1:160 speckled pattern, low titre aCL IgM | Arthritis | b and d: HCQ 200mg | 21.3 | - | 33 | 38+5 | PROM | 3065 | Intervillous thrombosis, perivillous fibrin deposits, decidual arteriopathy |
| 2* | UCTD | 4 | ANA 1:160 speckled pattern | Photosensitivity | b and d: HCQ 200mg  d: LMWH, ASA,  insulin,  levothyroxine | 25.3 | Atrophic gastritis; Hashimoto’s thyroiditis,  GDM | 38 | 36+3 | Preterm birth | 2540 | Signs of placental abruption, focal dystrophic calcifications |
| 3* | UCTD | 2 | ANA 1:640 homogeneous pattern | Arthritis, aphthosis | - | 27 | - | 25 | 40+6 | - | 3760 | Villous dysmaturity and chorangiosis, dystrophic calcifications |
| 4* | UCTD | 5 | ANA 1:80 nucleolar pattern, medium titre aCL IgG | Arthritis, thrombocytopenia | d: sulphasalazine, PDN 5 mg | 32 | Hashimoto’s thyroiditis | 29 | 41 | PROM | 3970 | Acute chorioamnionitis, cord phlebitis, vascular focal ectasia, stromal hyperplasia, perivillous and villous fibrin deposits, chronic deciduitis |
| 5* | UCTD | 5 | ANA 1:320 homogeneous pattern | Photosensitivity | d: HCQ 200mg, PDN 5 mg, ASA | 22.4 | 3 previous miscarriages, 1 VIP | 34 | 39+6 | - | 3190 | Focal chorioamnionitis, cord phebitis, chorionic vasculitis, perivillous and villous fibrin deposition, chronic deciduitis, decidual artheriopathy |
| 6* | UCTD | 0 | ANA:160 speckled pattern | Raynaud’s phenomenon, aphthosis | b and d: HCQ 200mg | 21 | - | 26 | 39+2 | - | 3180 | Perivillous and villous fibrin deposits, dystrophic calcifications, chronic deciduitis |
| 7 | UCTD | 2 | ANA:160 speckled pattern,  low titer aCL IgM | Arthritis, hypocomplementemia | b and d: HCQ 200mg, PDN 5 mg.  d: levothyroxine, ASA | 24.2 | Gestational hypothyroidism | 29 | 40+2 | - | 3530 | Villous chorangiosis, transmural infarction, avascular villi, vasculo-stromal cariorexis |
| 8 | UCTD | 1 | ANA 1:160 speckled pattern | Photosensitivity | - | 22.8 | MTHFR mutation (HZ) | 33 | 26+5 | Preterm birth | 601 | Mild-moderate acute chorioamnionitis, signs of placental abruption, intervillous thrombi,  perivillous and villous fibrin deposition |
| 9 | UCTD | 1 | ANA 1:160 speckled pattern | Photosensitivity | - | 22.8 | - | 33 | 26+5 | Preterm birth | 589 | Mild acute chorioamnionitis, signs of placental abruption, focal chronic deciduitis, perivillous and villous fibrin deposits |
| 10 | UCTD | 8 | ANA 1:160 speckled pattern | Arthralgia, Raynaud’s phenomenon | d:  alphamethyldopa | 29 | Basedow's disease;  GH, GDM | 40 | 34 | Preeclampsia, preterm birth | 2040 | Mild chronic villous hypoxic signs, villous infarction, fibrinoid deposits, decidual artheriopathy |
| 11* | SLE | 2 | ANA 1:160 homogeneous pattern, low titre aCL IgM and aβ2GPI IgG | Malar rash, Raynaud’s phenomenon, lymphopenia | b and d: HCQ  d: ASA, LMWH | 27 | - | 33 | 35+6 | Preterm birth | 2670 | Focal fibrin deposits, dystrophic calcifications |
| 12 | SLE | 6 | ANA 1:640 homogeneous pattern | Nephritis, leucopenia, arthritis | b and d: AZA 100mg, HCQ 200mg, PDN 7.5 mg, ASA | 22.6 | Previous preterm delivery | 29 | 35+1 | PROM, preterm birth | 2485 | Severe chronic hypoxic signs, syncytial knots,  villous agglutination, medium chronic deciduitis, decidual artheriopathy |
| 13 | SLE | 1 | ANA 1:640  Ro/SSA | Arthritis, photosensitivity, aphthosis, hypocomplementemia | b and d: HCQ 200mg, PDN 5 mg | 28 | - | 31 | 39+5 | Oligohydramnios | 2500, SGA | Signs of placental abruption, perivillous fibrin deposits |
| 14* | SjS | 4 | ANA1:160, Ro/SSA and La/SSB | Xerostomia, xeroftalmia | b and d: HCQ 200mg, levothyroxine | 23.2 | Hashimoto’s thyroiditis | 35 | 38+6 | PROM | 2890 | Villous hypovascolarization, dystrophic calcifications |
| 15 | IJA | 22 | ANA 1:80 nucleolar pattern | Arthritis | b and d: HCQ 200mg  d:  alphamethyldopa, ASA 100mg | 22 | GH | 33 | 35+6 | PROM, preterm birth | 2320 | Focal fibrin deposits, dystrophic calcifications |
| 16 | IJA | 19 | ANA 1:80 nucleolar pattern | Arthritis | b and d: HCQ 200 mg  d: ASA, alphamethyldopa, CCB | 24 | GH, proteinuria | 30 | 31 | Preeclampsia, IUGR, preterm birth | 1200 | Chronic villous hypoxic signs, chorangiosis, parenchimal infarction, subchorial and perivillous fibrin deposits |

BMI: body mass index. UCTD: undifferentiated connective tissue disease. ANA: antinuclear antibodies. aCL: anticardiolipin antibodies. HCQ: hydroxychloroquine. PROM: premature rupture of membranes. LMWH: low molecular weight heparin. GDM: gestational diabetes mellitus. ASA: acetylsalicylic acid 100mg. PDN: prednisone. VIP: voluntary interruption of pregnancy. MTHFR: methylenetetrahydrofolate reductase mutation. HZ: homozygosis. GH: gestational hypertension. SLE: systemic lupus erythematosus. aβ2GPI: anti beta2glycoprotein I antibodies. AZA: azathioprine. SGA: small for gestational age. SjS: Sjögren’s syndrome. IJA: idiopathic juvenile arthritis. CCB: calcium channel blockers. IUGR: intrauterine growth restriction.

*Patient with both paraffin and frozen samples.

**Supplementary table 2. Characteristics of healthy controls (HC).**

| Patient | Therapy before/during (b/d) pregnancy | BMI | Comorbidities and risk factors | Age at conception | Gestational week at delivery | Obstetric complications | Newborn weight (gr) | Placental characteristics (descriptive) |
| --- | --- | --- | --- | --- | --- | --- | --- | --- |
| 1 | b and d: levothyroxine | 22 | Hashimoto’s thyroiditis | 32 | 30 | Preterm birth | 1495 | Mild acute chorioamnionitis, signs of placental abruption |
| 2 | b: progestinic | 32 | Placenta previa | 39 | 30 | Preterm birth | 1665 | Villous haemorrhage |
| 3 | d: LMWH | 28 | Factor II mutation (HZ), MTHFR mutation (HZ) | 38 | 38+4 | - | 2960 | Mild acute chorioamnionitis |
| 4* | - | 25 | - | 34 | 39 | PROM | 3055 | Chorioamnionitis, low grade multifocal villitis of unknown origin |
| 5* | b and d: levothyroxine | 33.3 | Hypothyroidism | 40 | 38+2 | - | 3280 | Chorangiosis, villous haemorrhage |
| 6* | - | 27.5 | - | 35 | 37+6 | - | 2920 | Intimal hyperplasia of chorio-allantoid vessels |
| 7* | - | 25.6 | - | 37 | 41+5 | - | 3945 | Acute chorioamnionitis, oedematous membranes |
| 8* | b and d: levothyroxine | 25.3 | Hypothyroidism | 29 | 41+2 | PROM | 3390 | Altered maternal vascularization, focal chorangiosis |
| 9* | - | 26 | - | 29 | 40+3 | - | 3070 | Acute chorioamnionitis with fetal inflammatory response |
| 10* | b and d: levothyroxine | 20.5 | Hypothyroidism | 38 | 39+3 | - | 3490 | Chronic lymphocytic decidual inflammation |
| 11* | - | 26 | - | 29 | 36+2 | PROM, preterm birth | 2575 | Acute chorionitis, oedematous membranes |
| 12 | - | 24.5 | - | 33 | 36+1 | Preterm birth | 2650 | Intimal hyperplasia of chorio-allantoid vessels, villous infarcts |
| 13 | - | 26 | - | 30 | 40+2 | - | 3935 | Low grade, multi-focal villitis of unknown origin, acute chorioamnionitis with fetal inflammatory response |
| 14 | - | 24.8 | - | 38 | 40+2 | PROM, | 3905 | Villous thrombi, organized infarcts, acute chorionamnionitis without fetal response |
| 15 | - | 27.6 | - | 29 | 26 | PROM, preterm birth | 805 | Acute chorioamnionitis with fetal inflammatory response, intervillous thrombi, signs of placental abruption |
| 16 | b: progestinic | 32 | Hypertension | 31 | 26 | PROM, preterm birth | 769 | Necrotizing acute chorioamnionitis with marked fetal inflammatory response |

BMI: body mass index. LMWH: low molecular weight heparin. HZ: homozygosis. MTHFR: methylenetetrahydrofolate reductase mutation. PROM: premature rupture of membranes.

*Patient with both paraffin and frozen samples.
