# Supplementary figures and images for: Role of placental inflammatory mediators and growth factors in patients with rheumatic diseases with a focus on systemic sclerosis
Source: Rheumatology (Oxford). 2020 Dec 13;60(7):3307–16. doi: 10.1093/rheumatology/keaa782 (PMC8516508; doi:10.1093/rheumatology/keaa782)

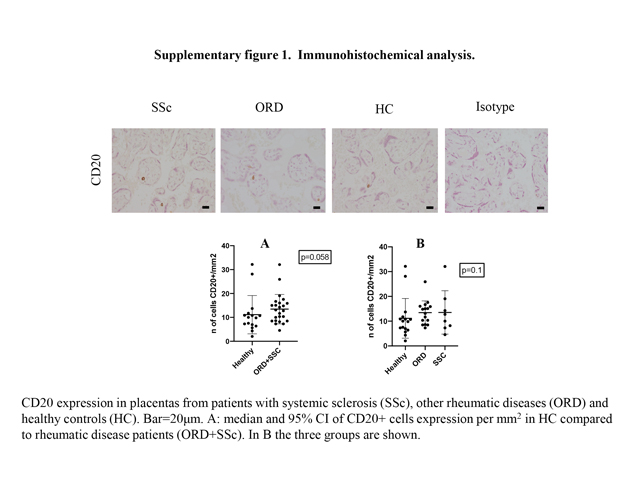

Supplement: keaa782_Supplementary_Data [file keaa782_Supplementary_Data.zip › rhe-20-1525-File006.jpg]

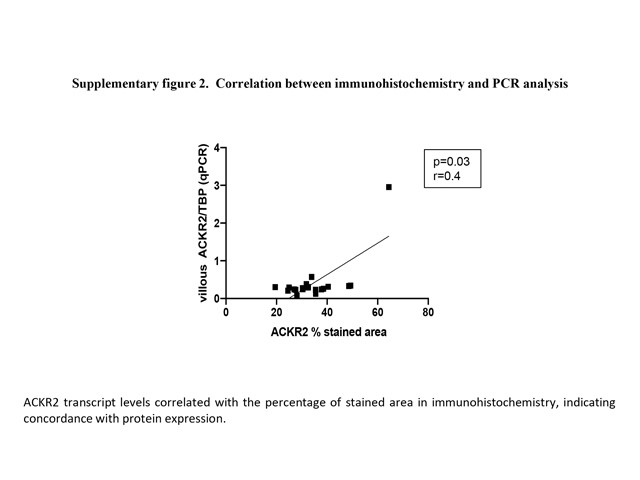

Supplement: keaa782_Supplementary_Data [file keaa782_Supplementary_Data.zip › rhe-20-1525-File007.jpg]

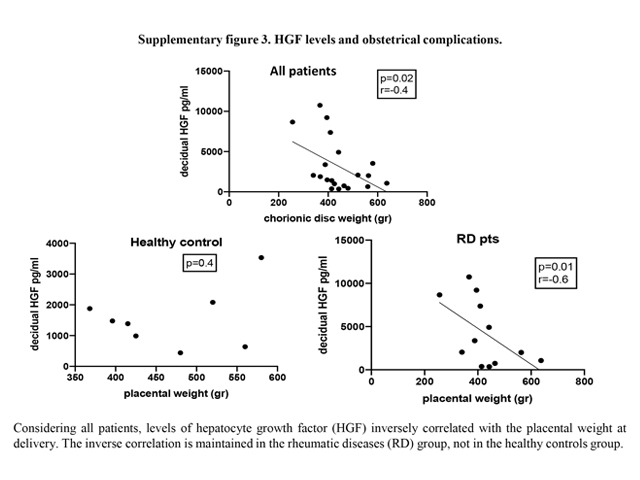

Supplement: keaa782_Supplementary_Data [file keaa782_Supplementary_Data.zip › rhe-20-1525-File008.jpg]
